# Bufalin‐Induced Epithelial‐to‐Mesenchymal Transition in Kidney Epithelial Cells

**DOI:** 10.1002/cbin.70091

**Published:** 2025-10-07

**Authors:** Gabriela Morais de Oliveira Barros, Kayo M. Bagri, Claudia Mermelstein, Luis Eduardo M. Quintas

**Affiliations:** ^1^ Instituto de Ciências Biomédicas Universidade Federal do Rio de Janeiro Rio de Janeiro Brazil

**Keywords:** bufadienolides, cell biology, Na^+^/K^+^‐ATPase, preclinical pharmacology, serial cell passage

## Abstract

The kidney plays a central role in fluid, electrolyte, and blood pressure regulation, processes tightly coupled to Na⁺/K⁺‐ATPase activity. Beyond its canonical transport function, Na⁺/K⁺‐ATPase also acts as a signaling receptor for cardiotonic steroids (CTSs) such as bufalin, which have been implicated in fibrosis and epithelial‐to‐mesenchymal transition (EMT). Here, we investigated the effects of serial passages on porcine kidney epithelial LLC‐PK1 cells and their response to the endogenous CTS bufalin. High‐passage cells (*P* > 80) displayed increased proliferation (1.7x), viability (1.5x), and migration (2.2x) compared to low‐passage cells (*P* < 40), concomitant with elevated ERK1/2 phosphorylation (2.5x), while NKA activity and expression remained unchanged. Bufalin treatment (20 nM, 48 h) induced striking morphological changes consistent with EMT in *P* > 80 cells, including a transition from cuboidal to elongated shapes with cytoplasmic extensions, whereas *P* < 40 cells were largely resistant. In high‐passage cells, bufalin reduced pan‐cadherin, E‐cadherin, occludin, claudin‐1, ZO‐1, and ZO‐2 expression, with redistribution of adhesion proteins from membrane to cytoplasm. β‐catenin and ZEB‐1 were excluded from the nucleus, indicating altered transcriptional regulation during EMT. In contrast, low‐passage cells exhibited only modest reductions in E‐cadherin, claudin‐1, and ZEB‐1, along with increased ZO‐2, and β‐catenin expression. For comparison, TGF‐β1 induced partial EMT features in bufalin‐resistant LLC‐PK1 cells, including striking cell elongation, increased vimentin expression, and appearance of E‐cadherin aggregates. Together, these results demonstrate that bufalin induces EMT‐like changes in LLC‐PK1 cells in a passage‐dependent manner, possibly through ERK1/2 activation, disruption of intercellular adhesion, and modulation of transcription factor localization. These findings highlight bufalin as a regulator of epithelial plasticity with potential implications for renal pathophysiology.

**Abbreviations**
ATP: adenosine triphosphate
BSA: bovine serum albumin
CKD: chronic kidney disease
CTS: cardiotonic steroids
DAPI: 4′,6‐diamidino‐2‐phenylindole
DMEM: Dulbecco's Modified Eagle Medium
DTT: dithiothreitol
EDTA: ethylenediaminetetraacetic acid
EGTA: ethylene glycol tetraacetic acid
EMT: epithelial‐mesenchymal transition
ERK1/2: extracellular signal‐regulated kinases 1/2
MTT: 3‐(4,5‐dimethyl‐2 thiazolyl)‐2,5‐diphenyl‐2H‐tetrazolium bromide
NKA: Na^+^/K^+^‐ATPase
PBS: phosphate buffered saline
Pi: inorganic phosphate
PMSF: phenylmethylfenyl fluoride
ZEB‐1: zinc finger E‐box binding homeobox 1

## 1 Introduction

Kidneys regulate body fluid, electrolytes, and acid–base balance. The organ is involved in the removal of metabolic waste and blood filtration, resulting in urine formation. Indeed, most of these tasks are performed by the nephrons, which are considered the functional units of the kidneys and are responsible for mechanisms of filtration, reabsorption, secretion, and urinary excretion. Nephrons also play an important regulatory role in blood pressure and modulate the transport of Na^+^ and water, particularly at the proximal convoluted tubule.

In kidney epithelial cells, the transmembrane protein Na^+^/K^+^‐ATPase (NKA) is localized at the basolateral membrane domain. The enzyme is a heteromeric protein, found in all mammalian cells, and is a member of an extensive family of P‐type ATPases highly conserved during evolution. It participates in electrolyte balance, fluid and ion transport, and is essential for cell survival. While it performs the active transmembrane transport of Na^+^ gradient leads to the reabsorption of this ion, thereby controlling natriuresis and diuresis (Blanco and Mercer 1998).

Cardiotonic steroids (CTSs) are natural compounds that selectively bind to the α subunit of NKA and inhibit its transport function. These steroids are categorized into two groups: those derived from vertebrates, such as bufalin and marinobufagenin, and those from plants, such as ouabain and digoxin (Akera and Brody 1977; Schoner and Scheiner‐Bobis 2007). Approximately 25 years ago, Xie and colleagues discovered that NKA also functions as a receptor for CTS, extending its physiological role beyond ion transport. Thus, when CTS binds to NKA that resides in caveolae, they trigger Src‐dependent signal transduction pathways involving various protein kinases and the production of second messengers (Liu and Xie 2010). Our group has demonstrated that CTSs, through activation of the NKA‐Src‐ERK1/2 cascade, induce natriuretic effects in isolated rat kidneys, possibly through endocytosis of the basolateral NKA in the tubular epithelium (Arnaud‐Batista et al. 2012; Godinho et al. 2017), and promote proliferation in cultured porcine kidney LLC‐PK1 cells (Amaral et al. 2018).

For several decades, the presence of endogenous CTS in mammals has been suggested as a regulator of blood pressure (Hamlyn and Blaustein 2017). Recently, evidence has emerged confirming this hypothesis. Both cardenolides and bufadienolides have been isolated and identified (Bagrov et al. 2009), though their biological functions remain unclear. What seems unequivocal is that CTSs reach higher concentrations in diseases of renal origin (Bagrov et al. 2009; Wang and Shapiro 2019). Bufadienolides such as telocinobufagin and marinobufagenin have been isolated from the plasma of patients with chronic renal failure (Komiyama et al. 2005), and bufalin from patients with cancer (Han et al. 2020).

Chronic kidney disease (CKD) is characterized by renal structural or functional modifications, accompanied by a decrease in glomerular filtration rate. A hallmark is interstitial fibrosis, highlighted by the activation and notable accumulation of fibroblasts and myofibroblasts, which are responsible for the production of the extracellular matrix. Renal fibrosis manifests through tubulointerstitial fibrosis, tubular atrophy, and glomerulosclerosis, compromising the usual architecture of the kidneys and ultimately resulting in the progression to end‐stage renal disease (He et al. 2013; Wang et al. 2021). One of the mechanisms responsible for fibrosis is epithelial‐to‐mesenchymal transition (EMT), which leads to cellular phenotypic changes, including the loss of cell‐cell adhesion, loss of cellular polarity, and the acquisition of invasive and migratory properties (Stone et al. 2016). EMT influences essential cellular processes such as gene regulation, cell cycle, adhesion, migration, cell signaling, differentiation, and cell death (Carvalho Leão et al. 2023).

Previous studies have shown that low nanomolar concentrations of bufalin enhance endocytosis of membrane proteins in NT2 cells (Rosen et al. 2004) and loss of intercellular adhesion and morphological changes (Feldmann et al. 2007). Also, marinobufagenin was found to stimulate collagen synthesis in human dermal fibroblasts (El‐Okdi et al. 2008). Fedorova et al. (2009) suggested that marinobufagenin promotes EMT in renal tubular epithelium. In rats infused with marinobufagenin for 4 weeks, there was an accumulation of cortical peritubular renal type I collagen and expression of the pro‐fibrotic transcription factor Snail, but epithelial markers E‐cadherin and β‐catenin were not altered. In LLC‐PK1 cells, morphological changes were observed after treatment with 100 nM of marinobufagenin for at least 72 h, but not with 100 nM of ouabain, and a reduction in the epithelial marker claudin‐1 (but not in E‐cadherin and occludin), along with an increase in the mesenchymal marker's vimentin, fibronectin, and type I collagen (Fedorova et al. 2009).

In this study, we investigated more deeply some characteristics of LLC‐PK1 renal cells with different in vitro passages, and the morphological and molecular changes induced by the endogenous CTS.

## 2 Materials and Methods

### 2.1 Antibodies and Fluorescent Probes

The following antibodies were used in this study: α‐tubulin (T‐9026), β‐catenin (A54441), laminin (L‐9393), and pan‐cadherin (C‐3678), from Sigma‐Aldrich (USA); claudin‐1 (D3H7C #13995), occludin (6B8A3 #68534), ZEB1 (#3396), ZO‐1 (D6L1E #13663), ZO‐2 (#2847), Twist1 (ESG9Y), N‐cadherin (13A9), and vimentin (D21H3), from Cell Signaling (USA); and E‐cadherin (610181) from BD Transduction Laboratories (USA). Secondary antibodies FITC‐labeled anti‐rabbit IgG (F‐0511), and FITC‐labeled anti‐mouse IgG (F‐5262 Fab specific), from Sigma‐Aldrich, USA; and affinity‐purified peroxidase‐labeled anti‐rat IgG (H + L) (Kirkegaard & Perry Laboratories, USA; Cat. No. 414‐1612). The DNA‐binding probe 4.6‐diamino‐2‐phenylindole dihydrochloride (DAPI) (D‐3571) and Texas Red‐phalloidin (T‐7471), Alexa 488, and DyLight 594. Secondary antibodies were purchased from Molecular Probes (USA).

### 2.2 Cell Culture

LLC‐PK1 cell line obtained from ATCC (CL‐101™, American Type Culture Collection, USA) was originally isolated from the proximal tubule of the kidney of Hampshire pig (*Sus scrofa domesticus*, male 3–4 weeks old). LLC‐PK1 cells (passages up to 40 ‐ *P* < 40 ‐, and higher than 80 ‐ *P* > 80) were cultured in 60 mm Petri dish (Jet Biofil, China) in Dulbecco's Modified Eagle's Medium (DMEM low glucose; Gilbco, USA) supplemented with 44 mM NaHCO_3_ (Sigma‐Aldrich, USA), 4.4 mM glucose (Sigma‐Aldrich, USA), containing 1% penicillin with 10.000 U streptomycin/mL (Gibco, USA), pH 7.2, enriched with 5% fetal bovine serum (FBS; Gibco, USA). Cells were grown in a humidified 5% CO_2_ atmosphere at 37°C for 3–4 days, and the medium was changed every 2 days.

### 2.3 Cell Counting and Proliferation

To assess the proliferative profile of LLC‐PK1 cells at *P* < 40 and *P* > 80, two 12‐well plates (Jet Biofil/TCP, China) were seeded with an initial density of 5.0 × 10^3^ cells per well in DMEM with 5% FBS and incubated for 24 h. For cell quantification, 3 wells of *P* < 40 and *P* > 80 were rinsed with 500 μL of sterile 1 × PBS and then trypsinized with 250 μL of 2.5% trypsin without EDTA in PBS per well. The number of Trypan blue‐viable cells was counted in a Neubauer chamber (hemocytometer) daily over a period of 3 days using inverted phase‐contrast microscopy (Nikon TS100, Nikon, Japan) at 20× magnification (Amaral et al. 2018).

### 2.4 Cell Viability Assay

A colorimetric assay was performed using 3‐(4,5‐dimethyl‐2‐thiazolyl)‐2,5‐diphenyl‐2H‐tetrazolium bromide (MTT; Sigma‐Aldrich, USA), which indirectly measures cell viability by detecting the mitochondrial enzymatic activity of living cells in LLC‐PK1 cells at *P* < 40 and *P* > 80. These cells were cultured with an initial density of 1.0 × 10^3^ cells per well in DMEM with 5% FBS in 96‐well plates (Corning Inc., USA). After the first 24 h, for up to 72 h with a 24 h interval, 20 μL of a solution containing 5 mg of MTT, solubilized in 1 mL of sterile PBS, was added to each well. The plate was incubated for 4 h to allow the conversion of tetrazolium salt to formazan, which was solubilized by adding 200 μL dimethyl sulfoxide (DMSO; Sigma‐Aldrich, USA). The absorbance of the plate was read at 570 nm using a Sunrise plate reader (Tecan, Switzerland), and the optical density values were normalized to baseline values and presented as a percentage of control (Amaral et al. 2018).

### 2.5 Cell‐Based Scratch Assay

The in vitro scratch assay aims to evaluate cell migration by creating an artificial gap. LLC‐PK1 cells from both groups were cultured in 30 mm Petri dishes (Jet Biofil, China) in DMEM with 5% FBS and incubated until they reached approximately 80% confluence. The LLC‐PK1 cells were allowed to grow in monolayers for 24 h, and after this period, a vertical scratch was made on the plate using a sterile 200 μL pipette tip. Next, the DMEM with 5% FBS was removed, and the detached and suspended cells were eliminated by washing with 1000 μL of sterile PBS. After washing, DMEM without FBS containing 5 μg/mL mitomycin (Y0000378; Sigma‐Aldrich, USA), diluted 1:10, was added. Before acquiring the images, the plate was washed again with 1 mL of sterile PBS. The scratch closure was then monitored daily over a period of 48 h in four fields using a phase‐contrast microscope (Olympus IX71, Olympus America, USA), utilizing a marking on the plate as a reference, and the rate of groove closure was evaluated using the ImageJ software (adapted from Stelling et al. 2021).

### 2.6 Na⁺/K⁺‐ATPase Activity Assay

To assess NKA activity, LLC‐PK1 cells were subcultured in four 100‐mm plates for 1 week. Cells were then trypsinized, resuspended in 2 mL of DMEM supplemented with 5% FBS, and collected into 1.5 mL microtubes (Eppendorf, USA). The samples were centrifuged at 1000 rpm for 3 min at 4°C, and the resulting cell pellet was stored at −80°C until further processing.

For membrane fraction isolation, 1 mL of homogenization buffer (containing 250 mM sucrose, 1 mM EGTA, 1 mM EDTA, 0.5 mM DTT, 20 mM Tris‐HCl (pH 7.2), 1 mM PMSF, and 0.01% SDS) was added to the frozen pellet. The sample was resuspended and transferred to a Potter‐Elvehjem homogenizer, followed by adding 3 mL of homogenization buffer. The homogenization process consisted of 10 cycles of 1 min homogenization with 30 s intervals on ice. The homogenate was centrifuged at 10,500 *g* for 20 min at 4°C (Hitachi, Japan), and the supernatant was discarded. The pellet was then subjected to ultracentrifugation at 70,000 *g* for 1 h at 4°C (Beckman, USA). The final pellet was resuspended in 200 µL of homogenization buffer and stored at −80°C.

After protein determination by the Lowry method, NKA activity was determined using the colorimetric method of Fiske and Subbarow, previously established in our laboratory (Bettero et al. 2011). This technique quantifies inorganic phosphate (Pi) released during ATP hydrolysis. The assay was performed in a reaction mixture containing 50 µg of protein (diluted lysate in sucrose buffer) incubated with 100 mM NaCl, 3 mM MgCl_2_, 10 mM KCl, 3 mM Na_2_ATP, 2.5 mM EGTA, 10 mM NaN_3,_ and 20 mM maleate‐Tris buffer (pH 7.4, 37°C). After 1 h incubation at 37°C, the reaction was stopped by adding 1 mL of ice‐cold Fiske and Subbarow reagent. The ammonium molybdate in the reagent reacted with Pi to form a blue phosphomolybdate complex, whose absorbance was measured at 650 nm using a spectrophotometer. Baseline ATPase activity was determined under the same conditions, except KCl was omitted, and 1 mM ouabain was added to selectively inhibit NKA. The final Pi concentration was calculated by interpolating absorbance values with the standard curve using a linear regression equation (y = ax + b) and corrected for dilution factors.

### 2.7 Western Blot

LLC‐PK1 cells were cultured in 100 mm plates until reaching 80–100% confluence. They were then washed with 4 mL PBS, the culture medium was removed, and the cells were washed again with PBS before lysis with 1 mL modified radioimmunoassay (RIPA) buffer (50 mM Tris‐HCl, 150 mM NaCl, 1% NP‐40, 5% sodium deoxycholate, 1 mM EDTA, 1 mM Na₃VO₄, 1 mM NaF, 1 mM PMSF, and a protease inhibitor cocktail). Cells were scraped, and the lysates were centrifuged at 10,000 *g* for 10 min at 4°C. The supernatant was collected, protein concentration was determined, and samples were subjected to Western blot analysis.

Proteins were separated by 10% SDS‐PAGE. A molecular weight marker (Thermo Fisher Scientific, USA) was included for band comparison. After electrophoresis, proteins were transferred onto a nitrocellulose membrane using constant voltage, and they were blocked for 1 h in 5% skim milk (Molico®) diluted in TTBS (TBS with 1% Tween‐20) and then incubated overnight at 4°C with primary antibodies against NKA α1 isoform (monoclonal; 1:1000 dilution; Invitrogen, USA) or phosphorylated and total ERK1/2 (polyclonal; 1:2000 dilution; Cell Signaling Technology, USA). After washing with TTBS, membranes were incubated for 1 h at room temperature under agitation with a secondary antibody (anti‐mouse IgG monoclonal or anti‐rabbit IgG polyclonal, 1:2000 dilution, Invitrogen, USA). Protein detection was performed using chemiluminescence reagents (Pierce™ ECL Western Blotting Substrate, Thermo Fisher Scientific, USA) and visualized with the ChemiDoc imaging system (Bio‐Rad, USA). Densitometric analysis was performed using ImageJ software (NIH, USA) to compare protein expression levels across experimental groups.

### 2.8 Phase Contrast Microscopy

LLC‐PK1 cells were grown in 6‐well plates (Jet Biofil/TCP, China), and upon reaching about 70% confluency, the DMEM + 5% FBS medium was removed, and the cells were deprived of FBS for 24 h for cell cycle synchronization. Subsequently, they were treated or not with 20 nM of bufalin (obtained by purification from the secretion of the *Rhinella schneideri* toad parotoid glands, as previously described ‐ Cunha‐Filho et al. 2010; Touza et al. 2011) and maintained for 48 h in a humidified 5% CO_2_ atmosphere at 37°C. For TGF‐β1, LLC‐PK1 cells were incubated in serum‐free DMEM for 6 h at 37°C to induce starvation. Subsequently, the medium was replaced with DMEM containing 1% FBS, with or without 4 ng/mL recombinant human TGF‐β1 (R&D Systems, USA), at a final concentration of. Cells were incubated for 72 h without medium replacement. Morphology of LLC‐PK1 cells treated with bufalin or TGF‐β1 was evaluated by Phase‐contrast microscopy (CellSens Software, Olympus IX71 Microscope, Olympus America, USA).

### 2.9 Immunofluorescence

LLC‐PK1 cells were cultured to 70% confluence in a 6‐well plate (Jet Biofil/TCP, China) on 22 mm‐Aclar plastic coverslips (Pro‐Plastics Inc., USA) previously coated with 0.1% gelatin, and subsequently treated with 20 nM bufalin for 48 h or 4 ng/mL TGF‐β1 for 72 h. Cells were washed three times with PBS to remove residual medium and then fixed with 4% paraformaldehyde in 0.1 M phosphate buffer for 15 min. Following fixation, cells were blocked with 3% BSA and 3% FBS in PBS for 1 h and then incubated overnight at 4°C in a humidified chamber with primary antibodies diluted 1:10 in PBS containing 3% BSA and 3% FBS. The next day, the cells were washed three times with PBS for 15 min each to remove excess primary antibody, followed by an additional blocking step with PBS containing 3% BSA and 3% FBS for 1 h. Secondary antibodies were then applied, and cells were incubated for 1 h at 37°C. Subsequently, cells were washed three times with PBS for 15 min each to remove excess primary antibody, followed by a final wash with 0.9% NaCl solution for 10 min. Nuclear staining was performed using DAPI fluorescent probe (1:2000) in 0.9% NaCl solution for 5 min. Phalloidin (1:100 in PBS) was used for the visualization of actin organization. Slides were mounted with Fluoromount aqueous mounting medium (Sigma‐Aldrich, USA # F4680), and images were randomly captured from six fields per condition using the Olympus FV1000 confocal microscope (Olympus America, USA) and CellSens 1.5 software. Control experiments with no primary antibodies showed only faint background staining (data not shown). Image processing was performed using Fiji software (Schindelin et al. 2012), and figure plates were mounted with Adobe Photoshop software (Adobe Systems Inc., USA).

### 2.10 Statistical Analysis

Statistical analysis was carried out using GraphPad Prism software version 8. The results of at least three independent experiments were compared. Statistical analysis was performed using Student's *t*‐test or two‐way ANOVA followed by Sidak's posttest (*p* > 0.05 was considered statistically significant). Data were expressed as means ± SEM.

## 3 Results

### 3.1 Effect of Serial Passages on the Proliferation of LLC‐PK1 Cells

**Figure 1:**
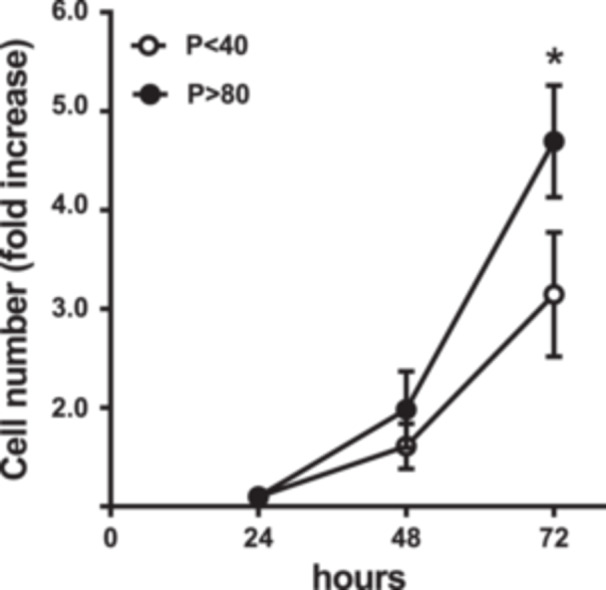
Evaluation of cell proliferation through the counting of LLC‐PK1 cells with low (*P* < 40) and high passages (*P* > 80) in the presence of 5% fetal bovine serum over a period of 24 to 72 h (*n* = 5). Data were expressed as means ± SEM. *p < 0.05 compared to *P* < 40.

The proliferative capacity evaluated by cell counting revealed that LLC‐PK1 *P* > 80 cells grew faster than *P* < 40 cells, resulting in a higher number of cells at the end of the experiment. As depicted in Figure 1, the number of *P* > 80 cells was 1.7‐fold higher when compared to *P* < 40 after 72 h.

### 3.2 Effect of Serial Passages on the Viability of LLC‐PK1 Cells

**Figure 2:**
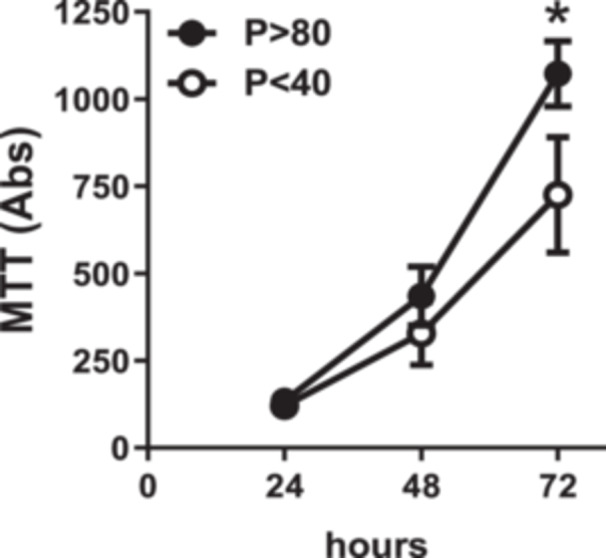
Evaluation of cell viability of LLC‐PK1 cells with low (*P* < 40) and high passages (*P* > 80) in the presence of 5% fetal bovine serum over period of 24 to 72 h (*n* = 6). Data were expressed as means ± SEM. *p < 0.05 compared to P < 40.

For the evaluation of cell viability, LLC‐PK1 *P* < 40 and *P* > 80 cells were subjected to the MTT assay at 24‐h intervals up to a 72 h‐period. It was observed that *P* > 80 cells had a significantly higher (almost 1.5‐fold) optical density than *P* < 40 cells after 72 h (Figure 2).

### 3.3 Effect of Serial Passages on the Migration Potential of LLC‐PK1 Cells

**Figure 3:**
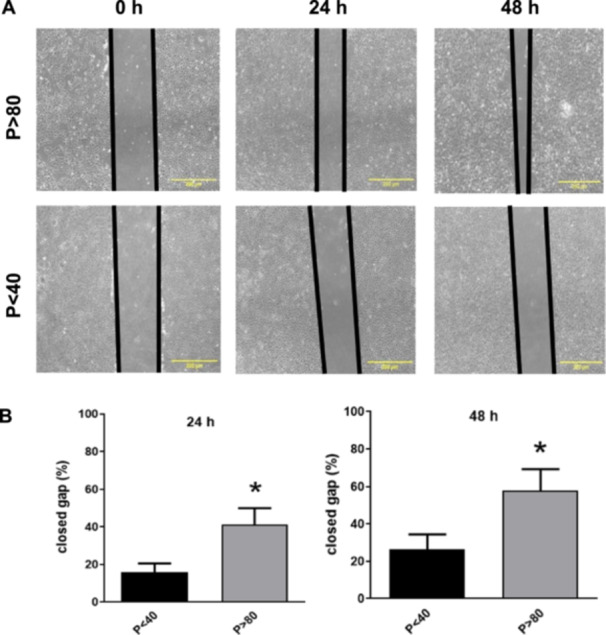
Evaluation of cell migration of LLC‐PK1 with high (*P* > 80) and low passages (*P* < 40) observed up to 48 h (*n* = 10) under Phase‐contrast microscopy (A). Scale bars = 200 µm. The images were analyzed using Image J software to evaluate the scratch by quantification of the closed gaps (B). The graphs show data expressed as means ± SEM. *p < 0.05 compared to *P* < 40.

The wound healing assay was initiated in vitro after creating a denuded area in a confluent culture. LLC‐PK1 *P* > 80 cells closed the open area faster and more efficiently than *P* < 40 cells (Figure 3). Indeed, after 24 h and 48 h LLC‐PK1 *P* > 80 cells exhibited a significantly higher level of cell migration (Figure 3).

### 3.4 Effect of Serial Passages on Na⁺/K⁺‐ATPase Activity and Protein Expression of LLC‐PK1 Cells

**Figure 4:**
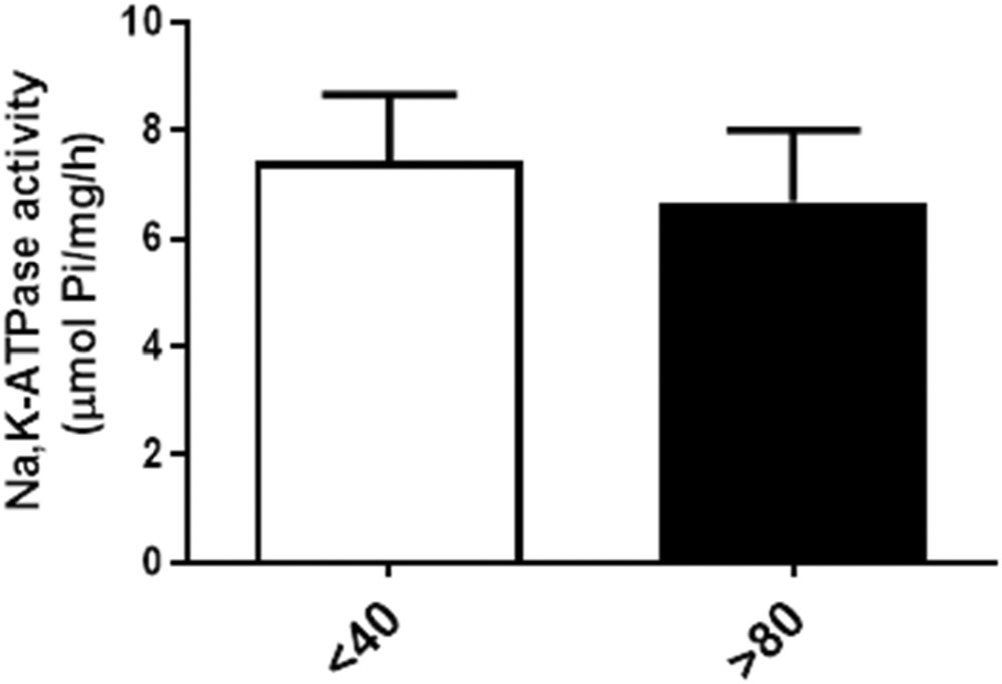
Na^+^/K^+^‐ATPase activity of LLC‐PK1 with high (*P* > 80) and low passages (*P* < 40). The graphs show data expressed as means ± SEM (*n* = 11).

**Figure 5:**
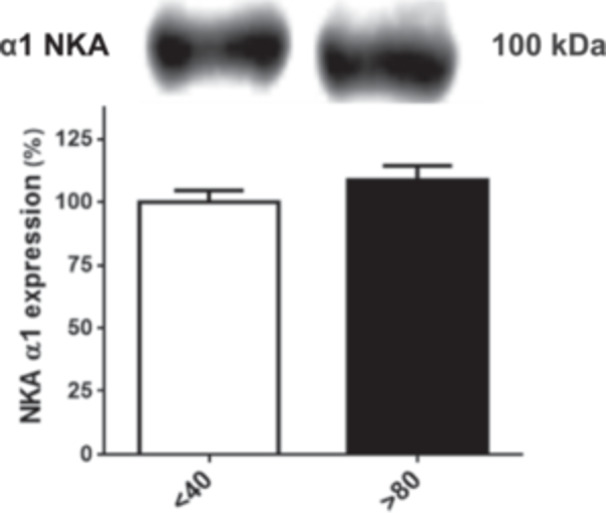
Relative expression of the Na⁺/K⁺‐ATPase α1 isoform in LLC‐PK1 cells with high (*P* > 80) and low passages (*P* < 40). The graphs show data expressed as means ± SEM. The Western blot band image shows a representative experiment (*n* = 4).

To investigate NKA activity, the primary molecular target of bufalin, membrane preparations were obtained from LLC‐PK1 cells at passages *P* < 40 and *P* > 80. Enzymatic activity was assessed using a colorimetric assay. As shown in Figure 4, no significant difference was observed between the two groups. Concordantly, no differences in NKA α1 protein expression were observed between the two groups, the only NKA isoform present in renal cells (Figure 5).

### 3.5 Effect of Serial Passages on Active and Total ERK1/2 Protein Expression in LLC‐PK1 Cells

**Figure 6:**
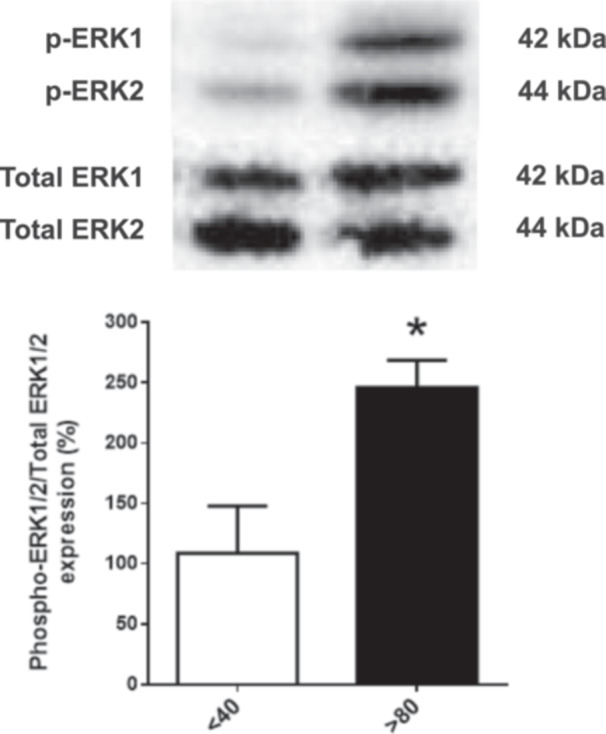
Relative expression of phosphorylated and total ERK1/2 in LLC‐PK1 cells with high (*P* > 80) and low passages (*P* < 40). The graphs show data expressed as means ± SEM. The Western blot band image shows a representative experiment (*n* = 3).

Given the role of ERK1/2 in cell viability and proliferation, we analyzed their expression in LLC‐PK1 *P* < 40 and *P* > 80 cells. An increased expression of the phosphorylated (active) form of ERK1/2 was observed in *P* > 80 cells compared to *P* < 40 cells, while the expression of total ERK1/2 remained unchanged. Consistently, this resulted in a significant increase in the p‐ERK/total ERK ratio (Figure 6).

### 3.6 Effect of Serial Passages on Bufalin‐Induced Alterations of LLC‐PK1 Cells Morphology

**Figure 7:**
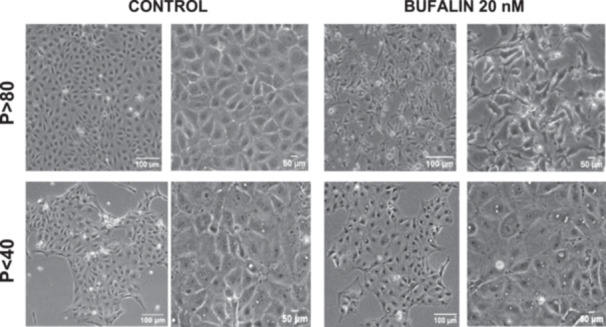
Phase‐contrast micrographs of LLC‐PK1 cells in the absence (control, left panels) and presence (treated, right panels) of 20 nM bufalin after 48 h. Scale bats = 50 µm or 100 µm.

LLC‐PK1 *P* < 40 and *P* > 80 cells were treated with 20 nM of bufalin for 48 h and were visualized using phase contrast microscopy. It was observed that the cell morphology of *P* < 40 cells was not affected by bufalin. In contrast, cell morphology was altered in *P* > 80 cells, consistent with EMT. Treatment of *P* > 80 cells with bufalin induced a change from a typical cuboidal epithelial cell shape rich in intercellular adhesions to a fusiform shape with the appearance of cytoplasmic extensions characteristic of mesenchymal cells (Figure 7).

### 3.7 Effect of Serial Passages on the Expression of EMT‐Related Proteins in Bufalin‐ and TGF‐β1‐induced Alterations of LLC‐PK1 Cells

**Figure 8:**
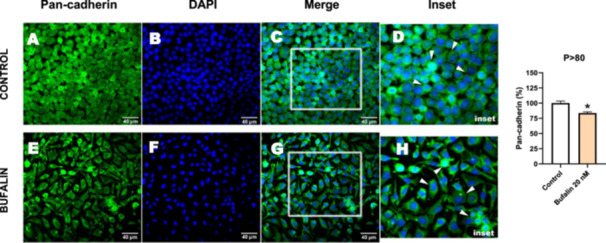
Bufalin slightly reduces pan‐cadherin expression in LLC‐PK1 P > 80 cells. Control cells (A) and bufalin‐treated cells (E) were stained with an antibody against pan‐cadherin (green) and DAPI for the visualization of nuclei (blue). Merged images are shown in C, G, D, and H. Images D and H show insets with higher magnification of the marked regions in C and G, where EMT can be observed in more detail (white arrowheads in D and H). Scale bars = 40 μm. Relative quantification of the fluorescence intensity of pan‐cadherin expression is depicted in the right panel. Data is expressed as means ± SEM. *p < 0.05 compared to control.

**Figure 9:**
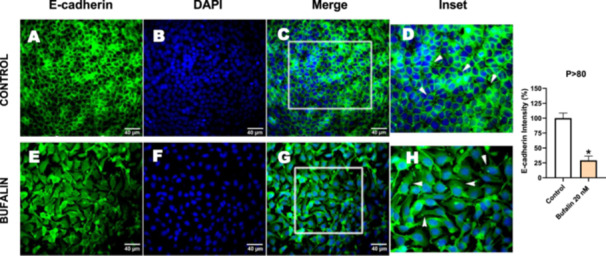
Bufalin reduces E‐cadherin expression in LLC‐PK1 P > 80 cells. Control cells (A) and bufalin‐treated cells (E) were stained with an antibody against E‐cadherin (green) and DAPI for the visualization of nuclei (blue). Merged images are shown in C, G, D, and H. Images D and H show insets with higher magnification of the marked regions in C and G, where EMT can be observed in more detail (white arrowheads in D and H). Scale bars = 40 μm. Relative quantification of the fluorescence intensity of E‐cadherin expression is depicted in the right panel. Data is expressed as means ± SEM. *p < 0.05 compared to control.

**Figure 10:**
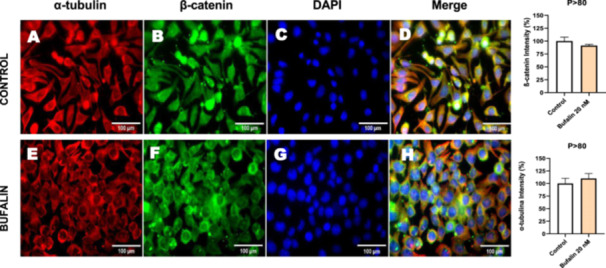
Bufalin does not modify α‐tubulin and β‐catenin expression in LLC‐PK1 P > 80 cells. Control cells (A and B) and bufalin‐treated cells (E and F) were stained with an antibody against α‐tubulin (red) and β‐catenin (green) and DAPI for the visualization of nuclei (blue). Merged images are shown in D and H. Scale bars = 100 μm. Relative quantification of the fluorescence intensity of α‐tubulin and β‐catenin expression is depicted in the right panel. Data is expressed as means ± SEM.

**Figure 11:**
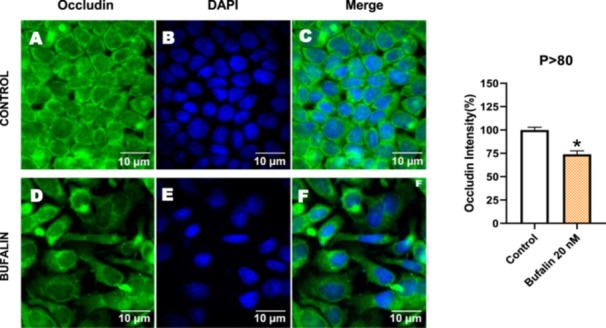
Bufalin reduces occludin expression in LLC‐PK1 P > 80 cells. Control cells (A) and bufalin‐treated cells (D) were stained with an antibody against occludin (green) and DAPI for the visualization of nuclei (blue). Merged images are shown in C and F. Scale bars = 10 μm. Relative quantification of the fluorescence intensity of occludin expression is depicted in the right panel. Data is expressed as means ± SEM. *p < 0.05 compared to control.

**Figure 12:**
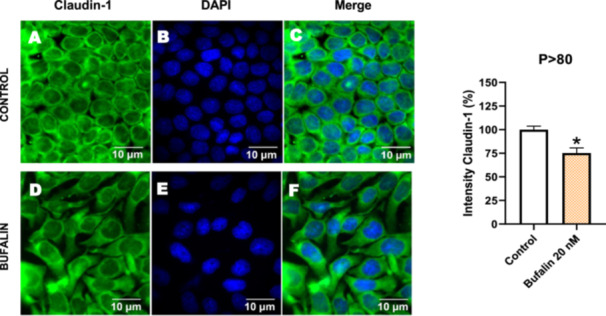
Bufalin reduces claudin‐1 expression in LLC‐PK1 P > 80 cells. Control cells (A) and bufalin‐treated cells (D) were stained with an antibody against claudin‐1 (green) and DAPI for the visualization of nuclei (blue). Merged images are shown in C and F. Scale bars = 10 μm. Relative quantification of the fluorescence intensity of claudin‐1 expression is depicted in the right panel. Data is expressed as means ± SEM. *p < 0.05 compared to control.

**Figure 13:**
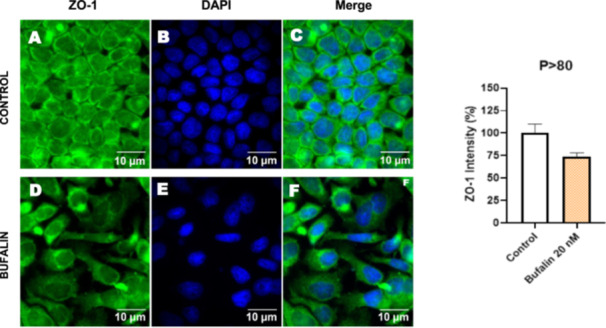
Bufalin reduces ZO‐1 expression in LLC‐PK1 P > 80 cells. Control cells (A) and bufalin‐treated cells (D) were stained with an antibody against ZO‐1 (green) and DAPI for the visualization of nuclei (blue). Merged images are shown in C and F. Scale bars = 10 μm. Relative quantification of the fluorescence intensity of ZO‐1 expression is depicted in the right panel. Data is expressed as means ± SEM. *p* = 0.0563 compared to control.

**Figure 14:**
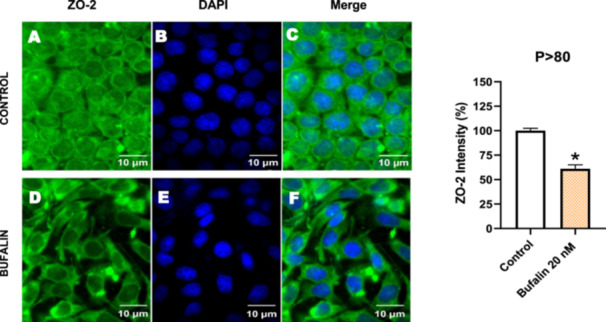
Bufalin reduces ZO‐2 expression in LLC‐PK1 P > 80 cells. Control cells (A) and bufalin‐treated cells (D) were stained with an antibody against ZO‐2 (green) and DAPI for the visualization of nuclei (blue). Merged images are shown in C and F. Scale bars = 10 μm. Relative quantification of the fluorescence intensity of ZO‐2 expression is depicted in the right panel. Data is expressed as means ± SEM. *p < 0.05 compared to control.

**Figure 15:**
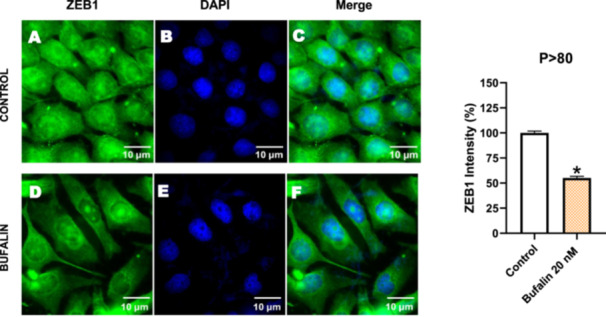
Bufalin reduces ZEB‐1 expression in LLC‐PK1 P > 80 cells. Control cells (A) and bufalin‐treated cells (D) were stained with an antibody against ZEB‐1 (green) and DAPI for the visualization of nuclei (blue). Merged images are shown in C and F. Scale bars = 10 μm. Relative quantification of the fluorescence intensity of ZEB‐1 expression is depicted in the right panel. Data is expressed as means ± SEM. *p < 0.05 compared to control.

Next, we investigated the distribution and expression of several EMT‐related proteins by immunofluorescence microscopy (Figures 8, 9, 10, 11, 12, 13, 14, 15). In *P* > 80 cells, bufalin treatment led to a reduction in pan‐cadherin (Figure 8) and E‐cadherin expression (Figure 9), accompanied by a redistribution of E‐cadherin from intercellular adhesion sites to the cytoplasm (Figure 9). Although overall β‐catenin expression was unchanged, its localization shifted markedly: while β‐catenin was nuclear in control cells, it was excluded from the nuclei of bufalin‐treated cells (Figure 10). The relative quantification of the nucleus/cytoplasm ratio of β‐catenin expression revealed a significant 30% decrease in treated cells. Tight junction proteins occludin (Figure 11), claudin‐1 (Figure 12), ZO‐1 (Figure 13), and ZO‐2 (Figure 14) were decreased and redistributed from the plasma membrane to the cytoplasm. Similarly, ZEB‐1 expression was reduced and relocalized from the nucleus to the cytoplasm (Figure 15). Together, these changes indicate that bufalin disrupts epithelial adhesion and barrier integrity in high‐passage cells, while also affecting the nuclear localization of transcriptional regulators.

**Figure 16:**
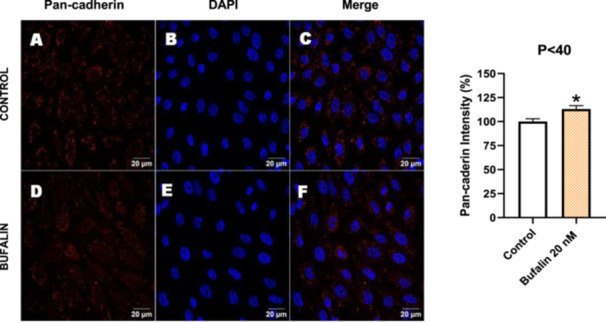
Bufalin slightly increases pan‐cadherin expression in LLC‐PK1 P < 40 cells. Control cells (A) and bufalin‐treated cells (D) were stained with an antibody against pan‐cadherin (red) and DAPI for the visualization of nuclei (blue). Merged images are shown in C and F. Scale bars = 20 μm. Relative quantification of the fluorescence intensity of pan‐cadherin expression is depicted in the right panel. Data is expressed as means ± SEM. *p < 0.05 compared to control.

**Figure 17:**
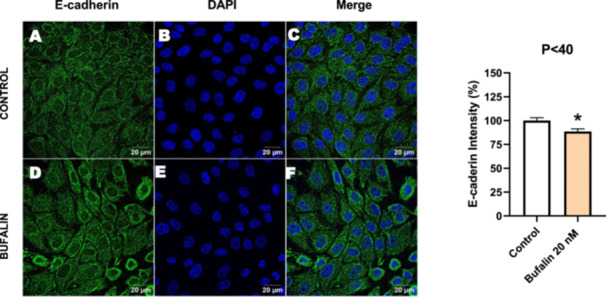
Bufalin slightly decreases E‐cadherin expression in LLC‐PK1 P < 40 cells. Control cells (A) and bufalin‐treated cells (D) were stained with an antibody against E‐cadherin (green) and DAPI for the visualization of nuclei (blue). Merged images are shown in C and F. Scale bars = 20 μm. Relative quantification of the fluorescence intensity of E‐cadherin expression is depicted in the right panel. Data is expressed as means ± SEM. *p < 0.05 compared to control.

**Figure 18:**
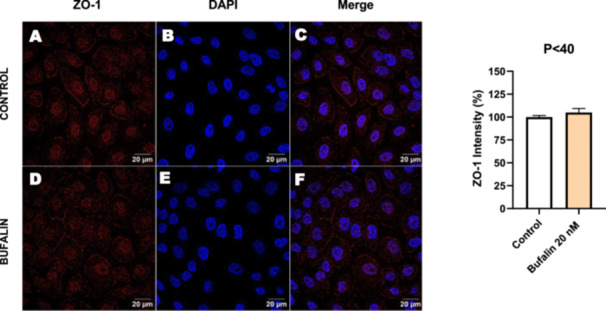
Bufalin does not modify ZO‐1 expression in LLC‐PK1 P < 40 cells. Control cells (A) and bufalin‐treated cells (D) were stained with an antibody against ZO‐1 (red) and DAPI for the visualization of nuclei (blue). Merged images are shown in C and F. Scale bars = 20 μm. Relative quantification of the fluorescence intensity of occludin expression is depicted in the right panel. Data is expressed as means ± SEM.

**Figure 19:**
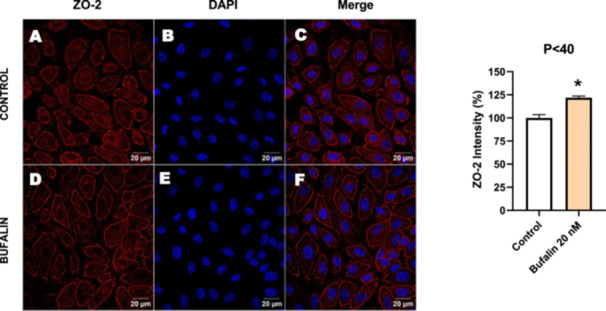
Bufalin increases ZO‐2 expression in LLC‐PK1 P < 40 cells. Control cells (A) and bufalin‐treated cells (D) were stained with an antibody against ZO‐2 (red) and DAPI for the visualization of nuclei (blue). Merged images are shown in C and F. Scale bars = 20 μm. Relative quantification of the fluorescence intensity of occludin expression is depicted in the right panel. Data is expressed as means ± SEM. *p < 0.05 compared to control.

**Figure 20:**
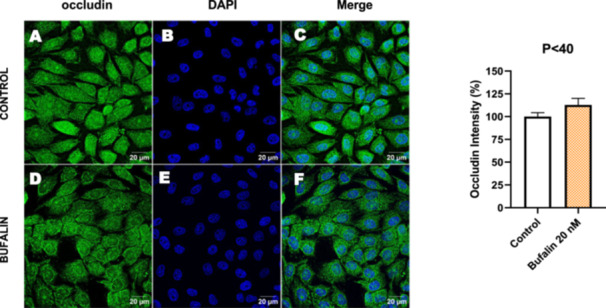
Bufalin does not modify occludin expression in LLC‐PK1 P < 40 cells. Control cells (A) and bufalin‐treated cells (D) were stained with an antibody against occludin (green) and DAPI for the visualization of nuclei (blue). Merged images are shown in C and F. Scale bars = 20 μm. Relative quantification of the fluorescence intensity of occludin expression is depicted in the right panel. Data is expressed as means ± SEM.

**Figure 21:**
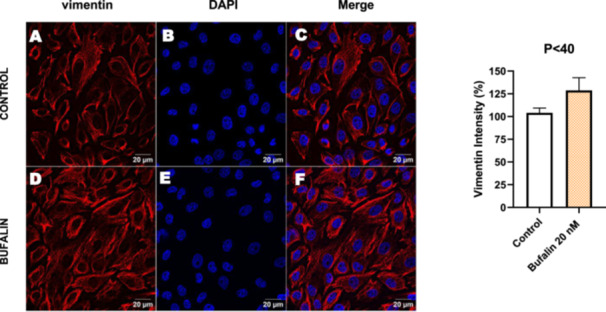
Bufalin does not modify vimentin expression in LLC‐PK1 P < 40 cells. Control cells (A) and bufalin‐treated cells (D) were stained with an antibody against vimentin (red) and DAPI for the visualization of nuclei (blue). Merged images are shown in C and F. Scale bars = 20 μm. Relative quantification of the fluorescence intensity of occludin expression is depicted in the right panel. Data is expressed as means ± SEM.

**Figure 22:**
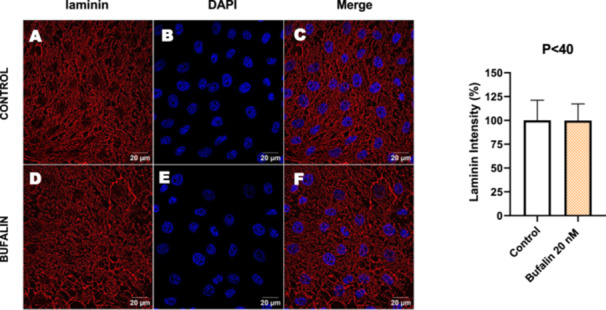
Bufalin does not modify laminin expression in LLC‐PK1 P < 40 cells. Control cells (A) and bufalin‐treated cells (D) were stained with an antibody against laminin (red) and DAPI for the visualization of nuclei (blue). Merged images are shown in C and F. Scale bars = 20 μm. Relative quantification of the fluorescence intensity of laminin expression is depicted in the right panel. Data is expressed as means ± SEM.

**Figure 23:**
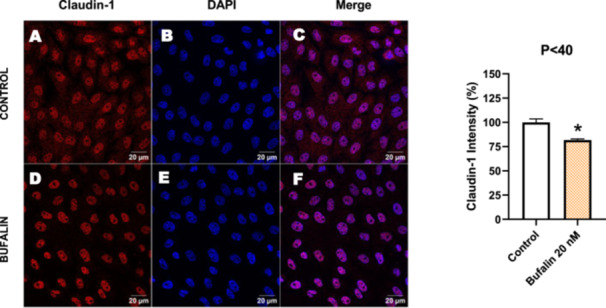
Bufalin reduces claudin‐1 expression in LLC‐PK1 P < 40 cells. Control cells (A) and bufalin‐treated cells (D) were stained with an antibody against claudin‐1 (red) and DAPI for the visualization of nuclei (blue). Merged images are shown in C and F. Scale bars = 20 μm. Relative quantification of the fluorescence intensity of claudin‐1 expression is depicted in the right panel. Data is expressed as means ± SEM. *p < 0.05 compared to control.

**Figure 24:**
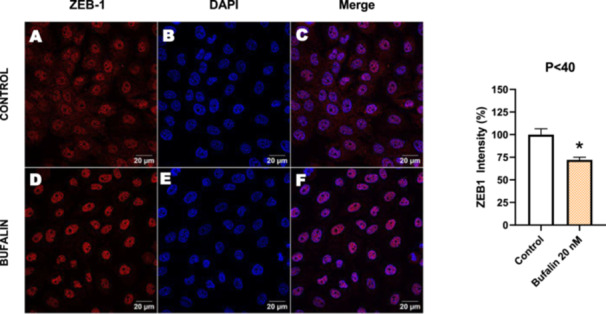
Bufalin reduces ZEB‐1 expression in LLC‐PK1 P < 40 cells. Control cells (A) and bufalin‐treated cells (D) were stained with an antibody against ZEB‐1 (red) and DAPI for the visualization of nuclei (blue). Merged images are shown in C and F. Scale bars = 20 μm. Relative quantification of the fluorescence intensity of occludin expression is depicted in the right panel. Data is expressed as means ± SEM. *p < 0.05 compared to control.

**Figure 25:**
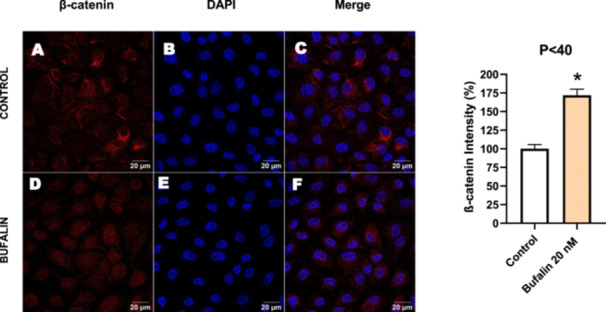
Bufalin increases β‐catenin expression in LLC‐PK1 P < 40 cells. Control cells (A) and bufalin‐treated cells (D) were stained with an antibody against β‐catenin (red) and DAPI for the visualization of nuclei (blue). Merged images are shown in C and F. Scale bars = 20 μm. Relative quantification of the fluorescence intensity of β‐catenin expression is depicted in the right panel. Data is expressed as means ± SEM. *p < 0.05 compared to control.

**Figure 26:**
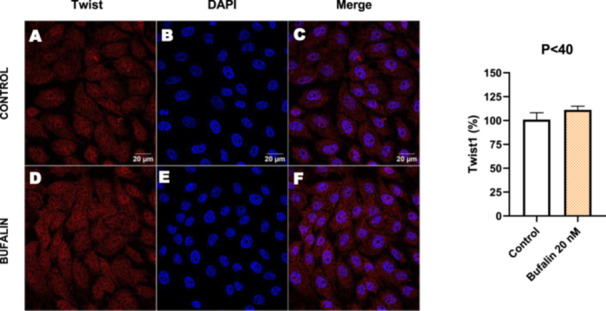
Bufalin does not modify Twist expression in LLC‐PK1 P < 40 cells. Control cells (A) and bufalin‐treated cells (D) were stained with an antibody against Twist (red) and DAPI for the visualization of nuclei (blue). Merged images are shown in C and F. Scale bars = 20 μm. Relative quantification of the fluorescence intensity of Twist expression is depicted in the right panel. Data is expressed as means ± SEM.

In contrast, *P* < 40 cells treated with bufalin showed very little or no significant alterations in pan‐cadherin (Figure 16), E‐cadherin (Figure 17), ZO‐1 (Figure 18), ZO‐2 (Figure 19), occludin (Figure 20), vimentin (Figure 21), laminin (Figure 22), claudin‐1 (Figure 23), and ZEB‐1 (Figure 24) expression, with increased β‐catenin expression that was redistributed from a perinuclear region (Figure 25), and ZEB‐1 (Figure 24) remained predominantly nuclear. There was no change in Twist expression (Figure 26). These features suggest negligible EMT‐like characteristics, denoted by some disruption of tight junctions and cytoskeletal remodeling, but with preserved epithelial adhesion.

**Figure 27:**
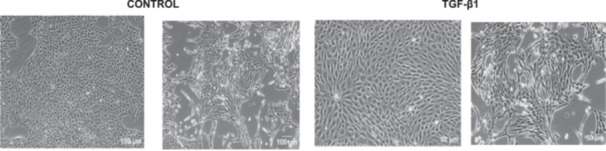
Phase contrast micrographs of LLC‐PK1 cells in the absence (control, left panels) and presence (treated, right panels) of 4 ng/mL TGF‐β1 after 72 h. Scale bars = 50 µm or 100 µm.

**Figure 28:**
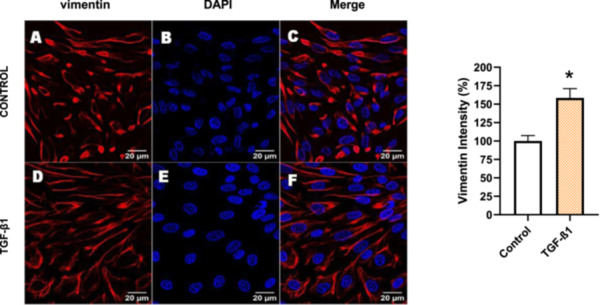
TGF‐β1 increases vimentin expression in bufalin‐resistant LLC‐PK1 cells. Control cells (A) and TGF‐β1‐treated cells (D) were stained with an antibody against vimentin (red) and DAPI for the visualization of nuclei (blue). Merged images are shown in C and F. Scale bars = 20 μm. Relative quantification of the fluorescence intensity of vimentin expression is depicted in the right panel. Data is expressed as means ± SEM. *p < 0.05 compared to control.

**Figure 29:**
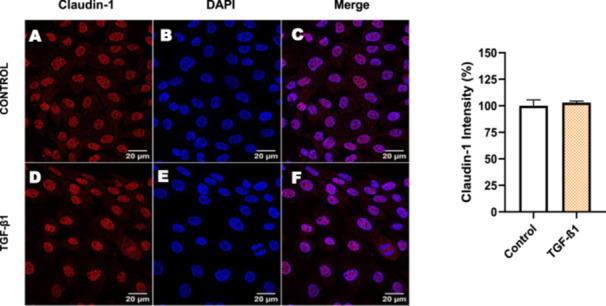
TGF‐β1 does not modify claudin‐1 expression in bufalin‐resistant LLC‐PK1 cells. Control cells (A) and TGF‐β1‐treated cells (D) were stained with an antibody against claudin‐1 (red) and DAPI for the visualization of nuclei (blue). Merged images are shown in C and F. Scale bars = 20 μm. Relative quantification of the fluorescence intensity of claudin‐1 expression is depicted in the right panel. Data is expressed as means ± SEM.

**Figure 30:**
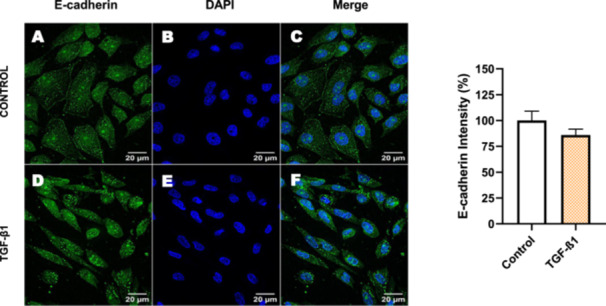
TGF‐β1 does not modify E‐cadherin expression in bufalin‐resistant LLC‐PK1 cells. Control cells (A) and TGF‐β1‐treated cells (D) were stained with an antibody against E‐cadherin (red) and DAPI for the visualization of nuclei (blue). Merged images are shown in C and F. Scale bars = 20 μm. Relative quantification of the fluorescence intensity of E‐cadherin expression is depicted in the right panel. Data is expressed as means ± SEM.

**Figure 31:**
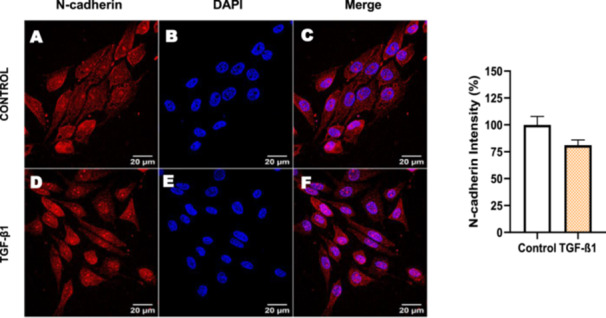
TGF‐β1 does not modify N‐cadherin expression in bufalin‐resistant LLC‐PK1 cells. Control cells (A) and TGF‐β1‐treated cells (D) were stained with an antibody against N‐cadherin (red) and DAPI for the visualization of nuclei (blue). Merged images are shown in C and F. Scale bars = 20 μm. Relative quantification of the fluorescence intensity of N‐cadherin expression is depicted in the right panel. Data is expressed as means ± SEM.

**Figure 32:**
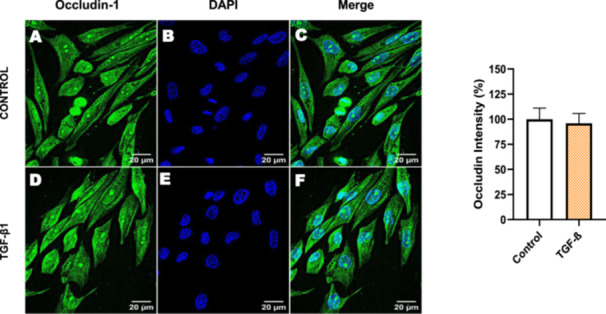
TGF‐β1 does not modify occludin‐1 expression in bufalin‐resistant LLC‐PK1 cells. Control cells (A) and TGF‐β1‐treated cells (D) were stained with an antibody against occludin‐1 (green) and DAPI for the visualization of nuclei (blue). Merged images are shown in C and F. Scale bars = 20 μm. Relative quantification of the fluorescence intensity of occludin‐1 expression is depicted in the right panel. Data is expressed as means ± SEM.

**Figure 33:**
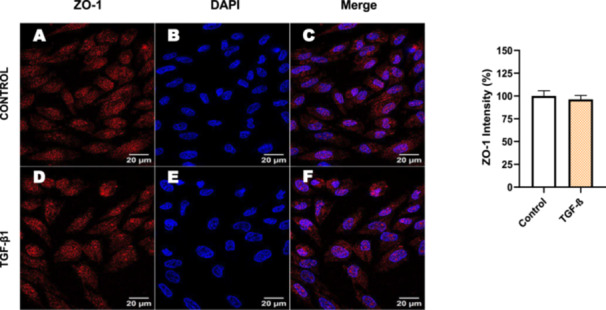
TGF‐β1 does not modify ZO‐1 expression in bufalin‐resistant LLC‐PK1 cells. Control cells (A) and TGF‐β1‐treated cells (D) were stained with an antibody against ZO‐1 (red) and DAPI for the visualization of nuclei (blue). Merged images are shown in C and F. Scale bars = 20 μm. Relative quantification of the fluorescence intensity of ZO‐1 expression is depicted in the right panel. Data is expressed as means ± SEM.

For comparison, LLC‐PK1 cells unresponsive to bufalin were treated with the classical EMT inducer TGF‐β1, as observed by Phase‐contrast microscopy, displayed a fusiform and elongated morphology, consistent with a mesenchymal‐like phenotype (Figure 27). The cells displayed a significant increase in vimentin staining (Figure 28), while treated cells appeared more spread than controls. Claudin‐1 expression remained unchanged (Figure 29), while E‐cadherin shifted to cellular aggregates (Figure 30), distinct from the cytoplasmic distribution observed after bufalin exposure (Figure 9). No differences were detected in N‐cadherin (Figure 31), occludin (Figure 32), or ZO‐1 (Figure 33) expression. These observations indicate that TGF‐β induced partial EMT‐associated alterations in low‐passage cells.

## 4 Discussion

Our study underscores the importance of considering serial passages in cell culture models, particularly when evaluating pharmacological responses. The observed differences between low‐passage (*P* < 40) and high‐passage (*P* > 80) LLC‐PK1 cells indicate that cell passage number is a critical variable influencing cellular behavior and response to external stimuli. Notably, high‐passage cells exhibited increased proliferative capacity, a higher migratory profile, and, importantly, were the only population exhibiting significant sensitivity to bufalin‐induced EMT since *P* > 40 cells barely responded to bufalin. ERK1/2, a protein kinase with a pivotal role in cell viability and proliferation, was more active in high‐passage LLC‐PK1 cells compared to low‐passage ones, potentially contributing to the observed differences in cellular behavior and response to bufalin.

Various cell lines are available as in vitro models for studying proximal tubular cells, making them a cost‐effective tool for basic research (Sánchez‐Romero et al. 2016 and 2020). We observed that LLC‐PK1 cells subjected to successive passages develop functional alterations, which are different from those found in cellular aging in mammalian cells (Blalock et al. 1999; Huang et al. 2001; Moon et al. 2003; Porter et al. 1997). LLC‐PK1 cells are widely used in toxicological studies for assessing various substances, including drugs, across different passage numbers. High‐passage cells, often exceeding 200 passages (Yoo et al. 1992; Liu et al. 1994; Morin et al. 1995; Vamvakas et al. 1996; Kruidering et al. 1998; Kevin k. divine, Felix Ayala‐Fierro 1999; Felix Ayala‐Fierro, dean e. Carter 2000; Kiyomiya et al. 2001; Machaalani et al. 2001; van de Water et al. 2001; Leussink et al. 2001, 2002; Blackmore et al. 2002; Gomes et al. 2002) are commonly utilized, though low‐passage cells, typically around 20 (Song et al. 2020) and 50 (Khundmiri et al. 2021) are also employed. Interestingly, high‐passage cells may exhibit a paradoxical increase in proliferative capacity, a phenomenon that has been documented in various other primary cell types, such as rat gastrointestinal mucosal cells (Atillasoy and Holt 1993; Xiao et al. 2001), vascular smooth muscle cells (VSMCs, McCaffrey et al. 1988; Vazquez‐Padron et al. 2004), and even infiltrating M1 macrophages and resident microglia following ischemic injury in the penumbra region (Moraga et al. 2015), where aging appears to drive an enhanced proliferation. Furthermore, in vitro proliferation increases approximately fivefold between neonatal and adult rats and nearly doubles between adult and aged rats (in contrast to fibroblasts from the same animals, Bochaton‐Piallat et al. 1993). Similar findings were observed in VSMCs aged in vitro, which exhibited nearly triple the growth rate (Moon et al. 2003). Immediately isolated VSMCs from aged rats contained similar levels of cytoskeletal proteins (α‐actin, desmin, myosin) as younger cells, but after 5 days in culture, α‐actin levels decreased, while desmin and myosin were no longer detectable, shifting towards a more synthetic phenotype (Bochaton‐Piallat et al. 1993). The type and, possibly, animal species can influence the behavior of cells (Stanulis‐Praeger 1987). Our results suggest that the proliferative feature seen in high‐passage LLC‐PK1 cells may be part of a broader phenomenon that extends beyond renal epithelial cell lines. Moreover, the enhanced migration observed in high‐passage cells supports the notion that these cells may exhibit a shift towards a more mesenchymal‐like phenotype, even in the absence of explicit morphological changes (Lamouille et al. 2014; Zohn et al. 2006; Ye et al. 2019).

Several alterations during serial passages have been described for epithelial cells. For instance, the transepithelial resistance of MDCK with 60–66 passages was much higher than with 110–116, despite having a smaller cell size, shorter and less dense microvilli, and a diverse glycolipid pattern (Barker and Simmons 1981; Nichols et al. 1986; Arthur 2000). For Caco‐2 cells, transepithelial resistance was reported to be lower (35–47 < 87–112 passages, Lu et al. 1996; 28–36 < 93–108 passages, Yu et al. 1997) or higher (16–19 > 89–99 passages, Artursson et al. 1993). Also, low‐passage cells have been reported to acquire a higher proliferation rate (Chantret et al. 1994; Lu et al. 1996), lower density, glucose consumption, hexose transporters, and more abundant brush border enzymes and paracellular transport (Yu et al. 1997; Mahraoui et al. 1992, 1994; Mesonero et al. 1994). A steady increase of Caco‐2 GLUT‐5 mRNA expression was observed after 26, 41, 66, 77, and 98 passages (Mahraoui et al. 1992). Renal proximal tubule opossum kidney (OK) cells with higher (53–74) compared to lower (52–65) passages are more prone to take up l‐DOPA, greater hydrogen peroxide levels as well as pro‐ (NADPH oxidase) and antioxidant (superoxide dismutase) enzymes, adhesion to plate surface, total protein expression and size (Gomes et al. 2002; Silva et al. 2006; Silva and Soares‐da‐Silva 2007, 2009) without changes in proliferation rate (Silva et al. 2006).

The primary molecular target of CTS is the plasma membrane NKA, and differences in bufalin sensitivity between the two LLC‐PK1 cell populations could stem from variations in pump activity or expression. However, our results indicate that serial passages did not impact these parameters. This aligns with multiple experimental studies reporting no significant changes in NKA activity within the renal cortex or proximal convoluted tubules of aged mice (Burich 1975) and rats (Beauchene et al. 1965; Bengele et al. 1983; Eiam‐Ong and Sabatini 1999; Silva et al. 2010), nor in NKA protein expression (Eiam‐Ong and Sabatini 1999; Silva et al. 2010). Conversely, Silva et al. (2006) and Silva and Soares‐da‐Silva (2011) observed increased NKA α1 expression and activity in OK cells clonal subpopulations. Discrepancies between these findings may be attributed to several factors, and, potentially, to spontaneously immortalized (OK or LLC‐PK1 cells, for instance) versus primary cells. More importantly, our data suggest that NKA activity and expression are unlikely to be directly responsible for the differential effects of bufalin.

A number of transcription factors act as inducers of EMT, inhibiting the expression of cell adhesion molecules and promoting epithelial cell reprogramming (Wynn 2008). The decreased expression and redistribution of molecules such as E‐cadherin and β‐catenin are classical markers of EMT. Previous studies have demonstrated that bufadienolides can induce renal fibrosis and EMT‐like processes. Fedorova et al. (2009) showed that chronic marinobufagenin administration evokes uremic cardiomyopathy and renal fibrosis in adult rats. Also, they found in LLC‐PK1 cell cultures that exposure to 100 nM over several days promotes EMT. However, only partial alterations of EMT markers were determined. Our data indicate that bufalin elicits a more rapid and pronounced EMT response in high‐passage LLC‐PK1 cells compared to low‐passage cells. In *P* > 80 cells, bufalin treatment led to a pronounced EMT‐like response characterized by loss of epithelial morphology, decreased expression of adhesion proteins such as pan‐cadherin and E‐cadherin, and redistribution of tight junction proteins, including occludin, claudin‐1, ZO‐1, and ZO‐2 from the plasma membrane to the cytoplasm. Importantly, β‐catenin localization shifted from the nucleus in control cells to the cytoplasm in bufalin‐treated cells. At the same time, ZEB‐1 expression was reduced and translocated from a nuclear to a cytoplasmic distribution. These changes indicate that bufalin disrupts epithelial adhesion and barrier integrity while altering the localization of EMT‐related transcriptional regulators in high‐passage cells.

Conversely, low‐passage cells (*P* < 40) exhibited a faint EMT phenotype after bufalin treatment, with very little to no modification of epithelial or mesenchymal biomarkers, except for an important increase in β‐catenin expression with perinuclear localization. The preservation of cadherins and nuclear ZEB‐1 in these cells indicates that epithelial identity is largely maintained, supporting the notion that cellular context and passage history critically shape the EMT response to bufalin.

Our results with bufalin‐resistant LLC‐PK1 cells exposed to TGF‐β1 revealed partial EMT‐like changes, as reflected by redistribution of E‐cadherin to membrane‐associated aggregates, and higher vimentin expression, while N‐cadherin, claudin‐1, occludin, and ZO‐1 remained largely unaffected. This pattern suggests that, in this cellular context, TGF‐β1 induces a clear morphological EMT program, but an incomplete framework of EMT biomarkers, altering adhesion and cytoskeletal markers without fully engaging the canonical cadherin switch.

Mechanistically, the stronger EMT‐like response in *P* > 80 cells may stem from passage‐dependent signaling differences, as these cells displayed elevated ERK1/2 phosphorylation, a pathway known to regulate proliferation, survival, and EMT. Bufalin may exploit this heightened ERK activity together with NKA endocytosis, destabilizing cell–cell adhesion and promoting redistribution of junctional proteins. At the same time, bufalin altered transcriptional regulation by excluding β‐catenin from the nucleus, thereby reducing Wnt‐driven EMT gene expression, and by shifting ZEB‐1 from the nucleus to the cytoplasm. This dual mechanism—junctional destabilization combined with attenuation of canonical EMT transcription factors—may explain the mixed EMT phenotype observed, in which adhesion loss and cytoskeletal remodeling occur without full activation of EMT‐associated transcriptional programs.

## 5 Conclusion

Taken together, our results suggest that bufalin acts as a conditional EMT modulator in kidney epithelial cells. In proliferative, high‐passage cells, bufalin disrupts adhesion complexes, alters transcription factor localization, and drives mesenchymal‐like remodeling. In low‐passage cells, the response is limited to modest junctional and cytoskeletal changes. These findings underscore the complexity of CTS signaling in renal epithelial cells and point to the possibility that endogenous bufadienolides may contribute to fibrosis and tissue remodeling in a context‐dependent manner, particularly under conditions of altered proliferative capacity or chronic kidney disease. The passage‐dependent variations observed in this study highlight the potential for notable biological and pharmacological differences that may impact data interpretation. As such, future research utilizing immortalized cell lines should incorporate standardized passage ranges to ensure the reproducibility and reliability of findings. These considerations are particularly relevant for toxicological assessments and preclinical studies investigating the effects of CTS on renal function.

## Author Contributions

**Gabriela Morais de Oliveira Barros:** conceptualization, investigation, data curation, formal analysis, methodology, validation, data curation, visualization, writing – original draft. **Kayo Moreira Bagri:** methodology, investigation, formal analysis, validation, visualization. **Claudia Mermelstein:** conceptualization, methodology, formal analysis, validation, writing – original draft, writing – review and editing, resources, funding acquisition, supervision. **Luis Eduardo M. Quintas:** conceptualization, methodology, formal analysis, validation, visualization, writing – original draft, writing – review and editing, resources, project administration, funding acquisition, supervision.

## Conflicts of Interest

The authors declare no conflicts of interest.

## Data Availability

The data that support the findings of this study are available from the corresponding author upon reasonable request.
